# Metagenomics of Ancient Fermentation Pits Used for the Production of Chinese Strong-Aroma Liquor

**DOI:** 10.1128/genomeA.01045-14

**Published:** 2014-10-23

**Authors:** Ming-Yi Guo, Dan-Qun Huo, Rohit Ghai, Francisco Rodriguez-Valera, Cai-Hong Shen, Niang Zhang, Su-Yi Zhang, Chang-Jun Hou

**Affiliations:** a College of Bioengineering, Chongqing University, Chongqing, China; b Evolutionary Genomics Group, Departamento de Producción Vegetal y Microbiología, Universidad Miguel Hernández, San Juan de Alicante, Alicante, Spain; c National Engineering Research Center of Solid-State Brewing, Luzhou Laojiao Group Co. Ltd, Luzhou, China

## Abstract

The complex microbiota of pit mud of solid-state fermentation reactors used for the production of Chinese liquor is responsible for producing one of the oldest distillates in the world. We apply a deep-sequencing approach to characterize the microbiota from pits that have been in use for up to 440 years.

## GENOME ANNOUNCEMENT

Strong aromatic Chinese liquor is produced by a traditional process of solid-state fermentation that has been in use for hundreds of years. The final product, with a high ethanol content (40% to 55%), is among the most consumed liquors in the world, with an annual consumption of ~4 billion liters (1). The most characteristic feature of production of this liquor is that the process is carried out in specialized rectangular soil pits, whose walls have been coated by a special mixture of fermentation mud made largely with clay and grain. The age of this coating, the microbiota of which is an important inoculum for fermentation, is believed to influence final liquor quality. Microbes involved in the process have been studied by cultivation-dependent and -independent methods (2–5). However, due to the limitation of the research approaches used, the microbial diversity is still poorly understood. This is the first report which describes a deep metagenomic sequencing-based study of the microbial diversity of 12 pits of various ages, ranging from comparatively young (50 years) to old (140 and 220 years) and extremely old pits, which have been in constant use for 440 years.

Mud samples were collected from the top, middle, and bottom of each pit (3 pits for each age) and DNA was extracted using the Power Soil DNA isolation kit (MoBio Laboratories, Inc., Carlsbad, CA, USA). Sequencing was performed using Illumina HiSeq 2000 (South-West University, Chongqing, China). A total of 4 Illumina lanes were sequenced for each age. All sequences were compared against the Ribosomal Database Project (6) to analyze and identify the 16S rRNA genes, and the entire data set was also annotated using the MG-RAST server (7).

We found significant differences in the community structure of the pit muds across the different ages. The microbial communities in all the pits were dominated by *Firmicutes*, which were present in similar proportions in the youngest and the oldest pits (57% and 51%, respectively) but in slightly higher proportions in the 140- and 220-year-old pits (70% and 73%, respectively). The youngest pit (50 years) also had the highest proportions of *Gammaproteobacteria* (8%) and opisthokonts (8%), whose populations were significantly reduced in all the other pits (>1%). Remarkably, the abundances of *Bacteroidetes* increased substantially in the oldest pit (440 years) to almost 14%, nearly double that of all other pits. A similar increase was also found for *Euryarchaeota*, which comprised nearly 22% of the community in the oldest pit relative to the others, where the proportions ranged from 13% to 14%.

This study suggests that the pit microbiota, though always dominated massively by *Firmicutes*, does show subtle changes in the overall composition, particularly in the increase of *Euryarchaeota* and *Bacteroidetes* as the age of the pit increases. Understanding the microbial diversity in the pits will help with the exploration of new microbial strains for food fermentation and improving the quality of Chinese liquor.

### Nucleotide sequence accession numbers.

The sequences of all metagenomes have been deposited at the NCBI Sequence Read Archive under the accession numbers SRX691263 to SRX691265 and SRX691272 to SRX691280.
